# Supplementary figures and images for: Dysregulated Immune Responses in COVID-19 Patients Correlating With Disease Severity and Invasive Oxygen Requirements
Source: Front Immunol. 2021 Oct 21;12:769059. doi: 10.3389/fimmu.2021.769059 (PMC8567168; doi:10.3389/fimmu.2021.769059)

Supplementary Figure 1

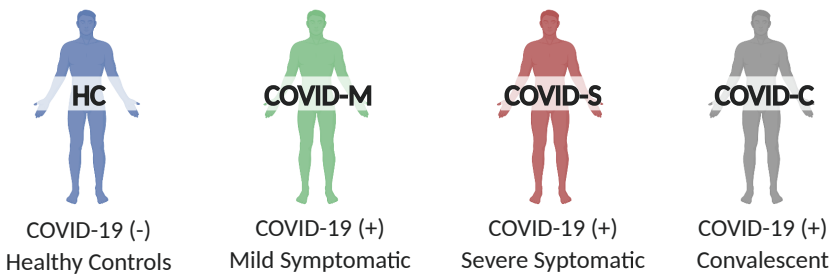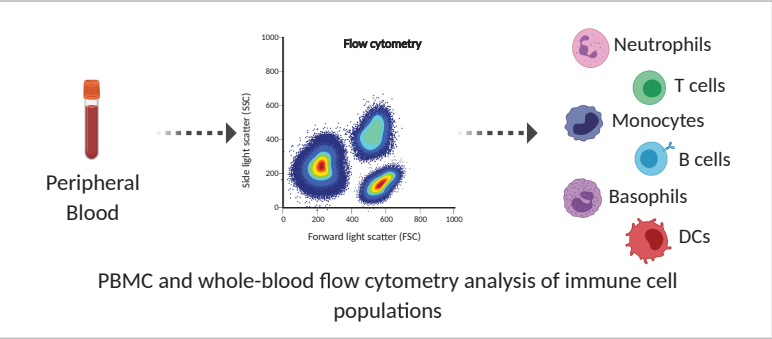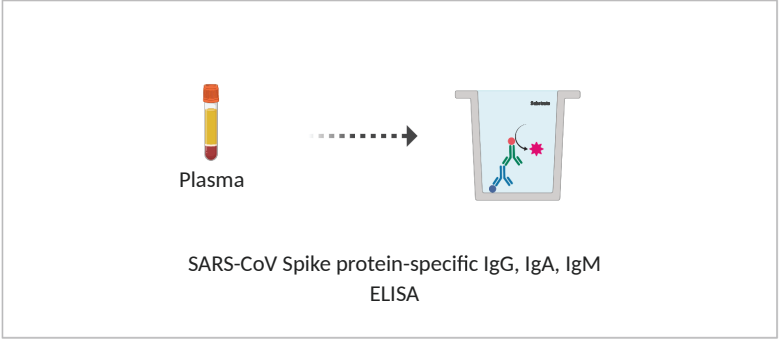

Supplement: Supplementary Figure 1 — COVID-19 study diagram. (A) In this study, 29 COVID-19 Moderate and 37 Severe COVID-19 patients, along with 28 convalescent patients and 28 Healthy controls were recruited from the Hospital Clínico Universidad de Chile for the study of the immunological landscape and disease evolution. [file DataSheet_1.pdf]

# Supplementary Figure 2

A.

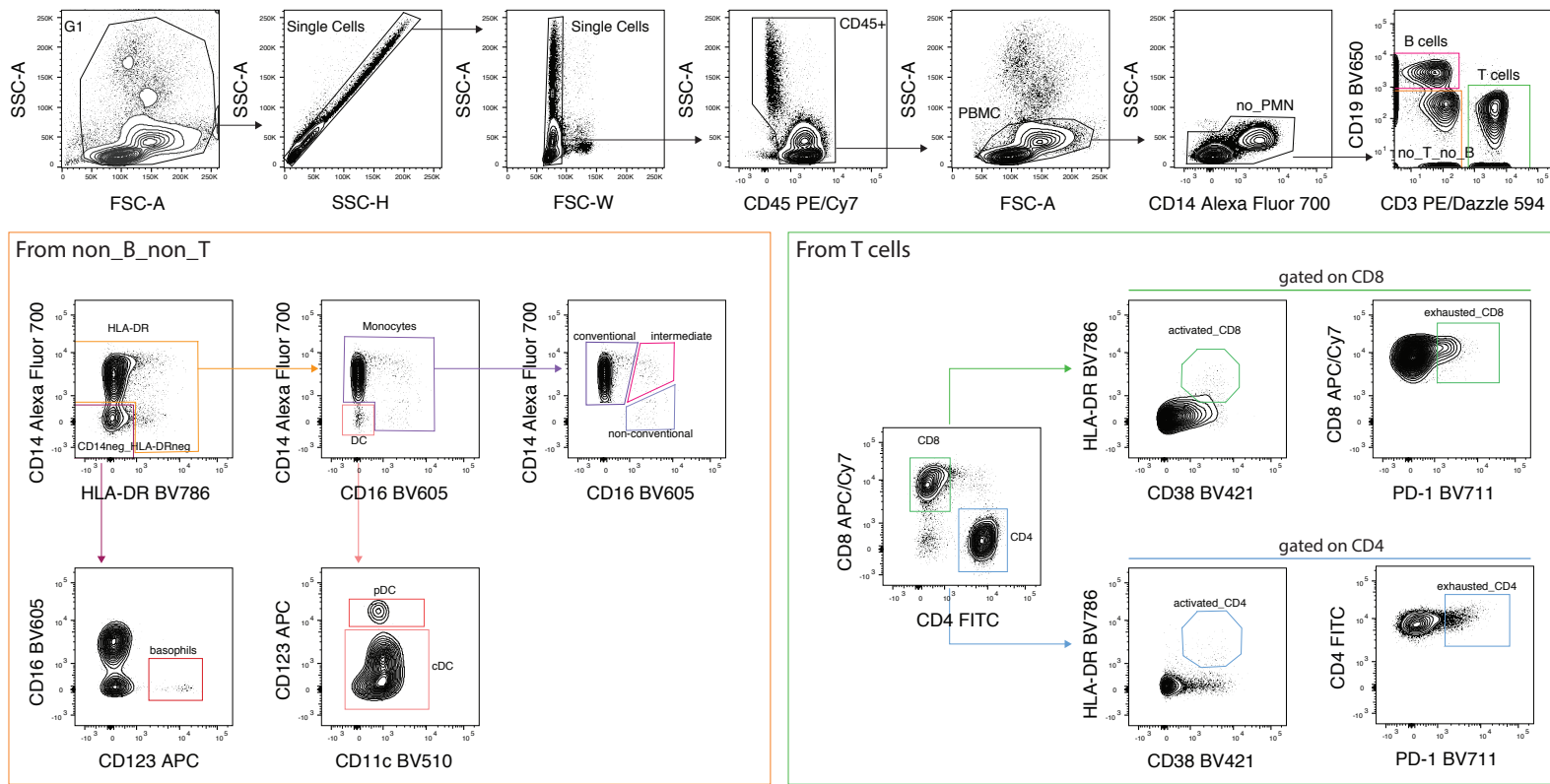

B.

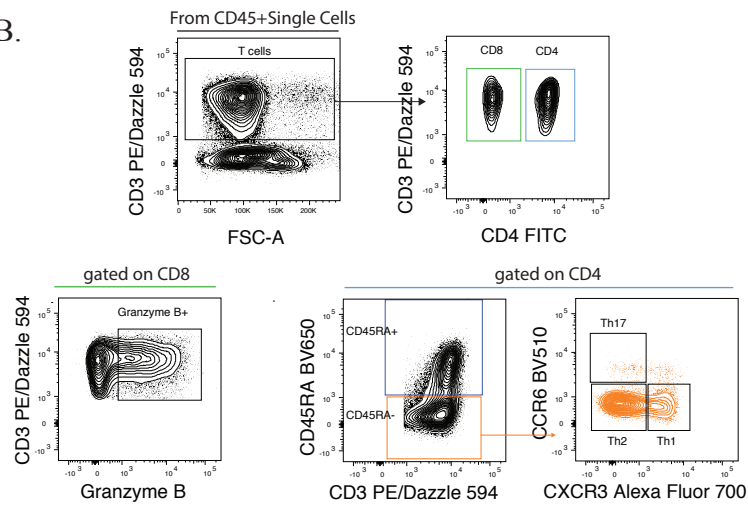

C.

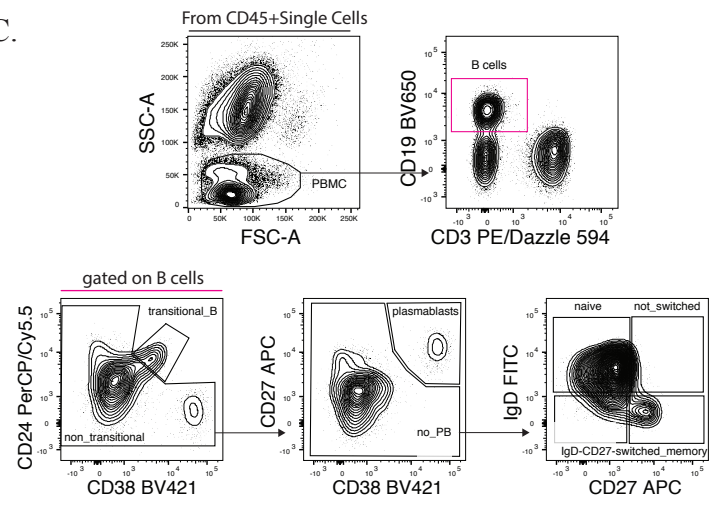

D.

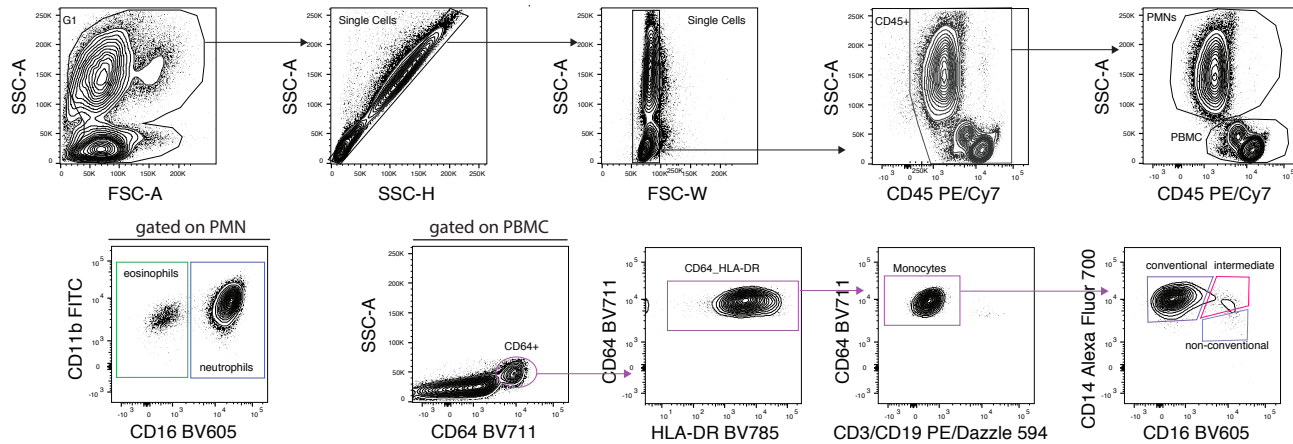

Supplement: Supplementary Figure 2 — Gating strategies used for flow cytometric analyses of immune cell subsets. (A) General analysis of main immune populations found on peripheral blood mononuclear cells of patients (B cells, CD8 and CD4 T cells, Monocytes, DCs and basophils). (B) Gating strategy used to examine CD8 and CD4+ T cell populations in peripheral blood of COVID-19 patients. (C) Gating strategy used to identify different B cell populations (Transitional, non-transitional, plasmablasts, näive, IgD-CD27- and memory switch B cells) in fresh blood samples. (D) Characterization of granulocytes and monocytes through flow cytometry analysis in fresh blood samples. [file DataSheet_2.pdf]

Supplementary Figure 3

A.

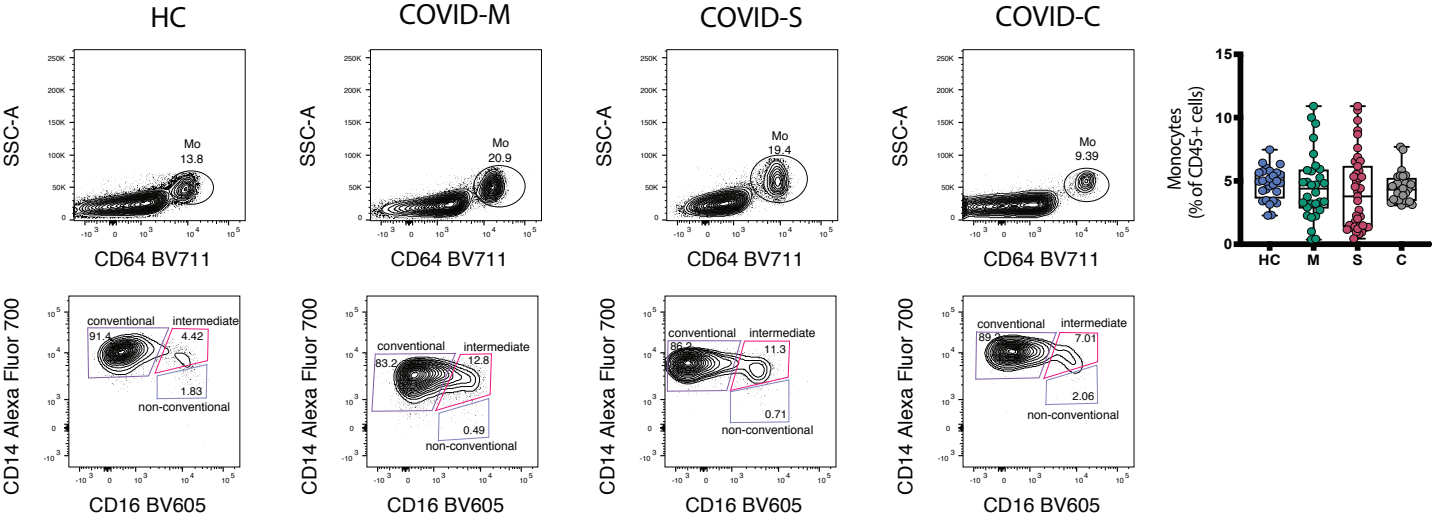

B.

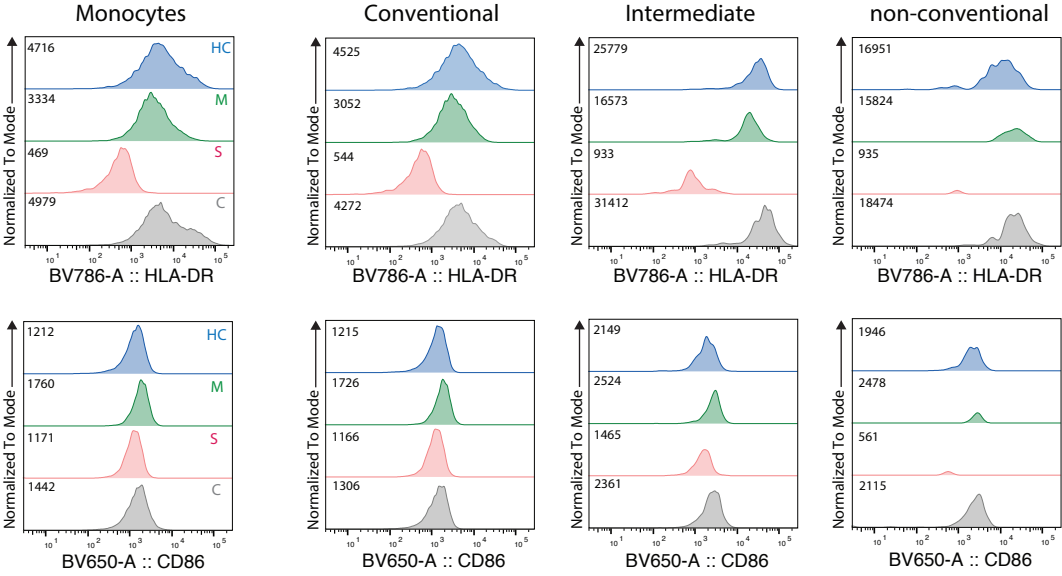

C.

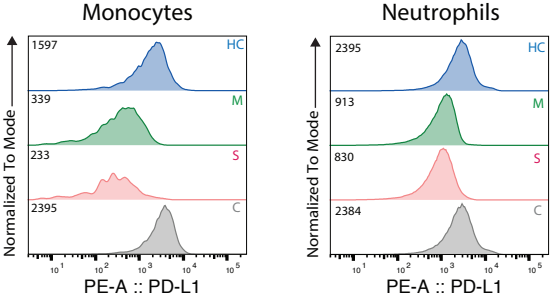

Supplement: Supplementary Figure 3 — Granulocyte identification and Monocyte characterization from peripheral blood of COVID-19 patients. (A) Representative facs plots and frequency of monocyte populations of all subjects included in the study: healthy controls (HC), moderate (M), severe (S) and convalescent (C) COVID-19 patients. (B) Representative histograms for HLA-DR and CD86 expression assessed in the different monocyte subsets (conventional, intermediate and non-conventional monocytes). (C) Representative histograms for PD-L1 expression determined in neutrophils and monocytes from all conditions studied (healthy controls (HC), moderate (M), severe (S) and convalescent (C) COVID-19 patients). [file DataSheet_3.pdf]

# Supplementary Figure 4

A.

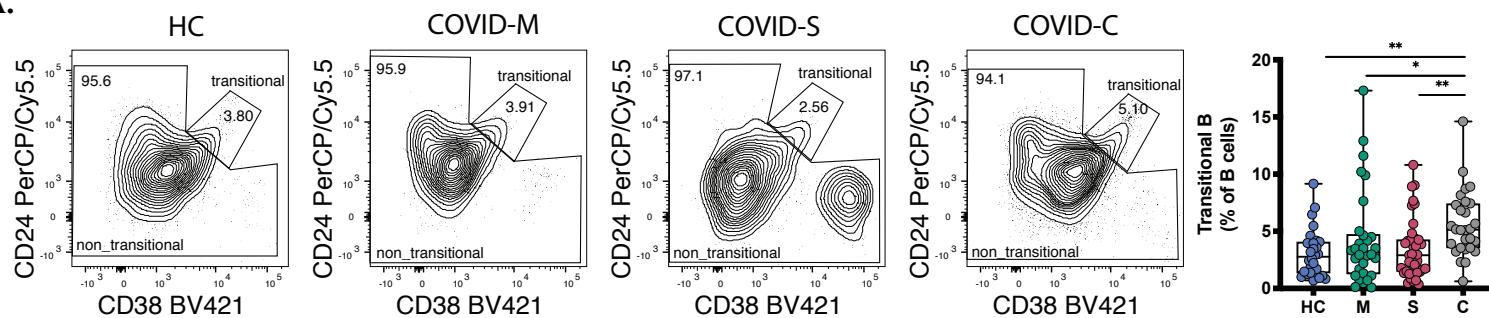

B.

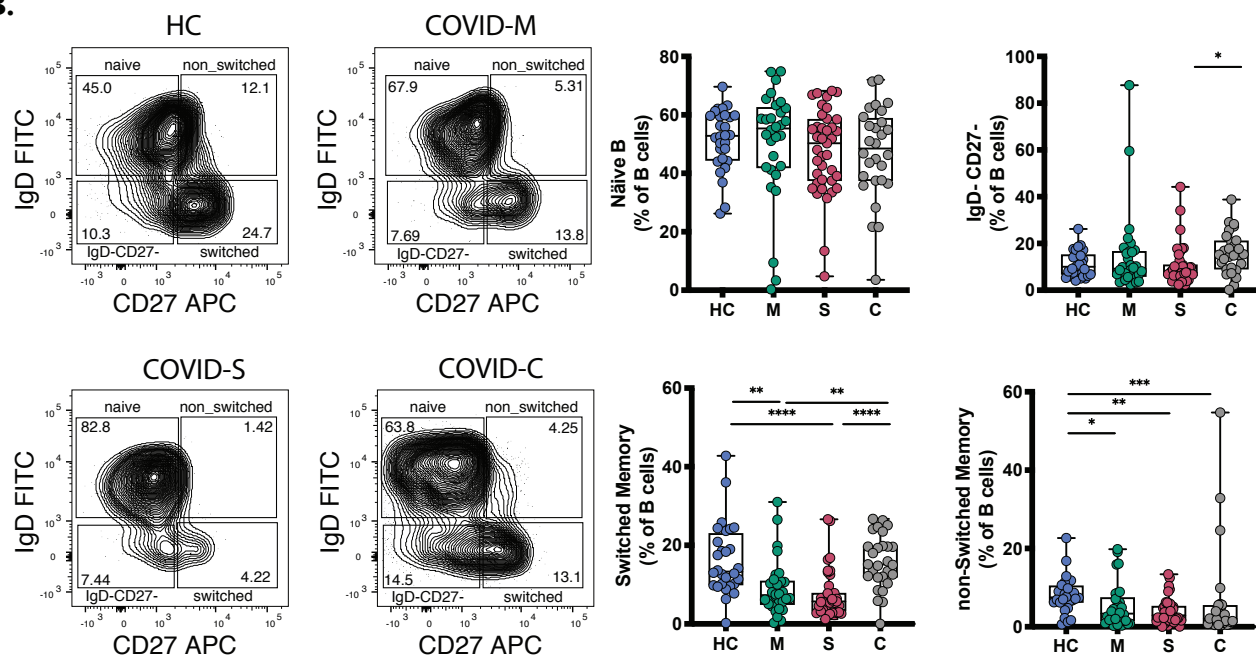

Supplement: Supplementary Figure 4 — Identification of cellular immune components in peripheral blood of COVID-19 patients. (A) Representative facs plots and frequency of transitional and non-transitional B cell populations for all conditions included in the study: healthy controls (HC), moderate (M), severe (S) and convalescent (C) COVID-19 patients. (B) Representative facs plots and frequency of näive, IgD-CD27- and memory switched B cells identified in all 4 subjects studied. (C) Representative facs plots and frequency of CCR6+CXCR3- Th17- equivalents characterized in all 4 conditions studied (healthy controls (HC), moderate (M), severe (S) and convalescent (C) COVID-19 patients). [file DataSheet_4.pdf]
